# Investigation of the effects of angiotensin converting enzyme inhibitors and angiotensin receptor blockers on anemia in patients with normal or mildly low glomerular filtration rate

**DOI:** 10.3906/sag-2104-138

**Published:** 2021-09-12

**Authors:** Betül ERGÜN, Aysun AYBAL KUTLUGÜN, Mustafa Çağrı ERGÜN, Galip AKTÜRK, Esin BEYAN

**Affiliations:** 1 Department of Internal Medicine, Health Science University, Keçioren Training and Research Hospital, Ankara, Turkey; 2 Division of Nephrology, Department of Internal Medicine, Health Science University, Keçiören Training and Research Hospital, Ankara, Turkey; 3 Division of Rheumatology, Deparrtment of Internal Medicine, Necmettin Erbakan University Faculty of Medicine, Konya, Turkey; 4 Department of Internal Medicine, Polatlı Duatepe Hospital, Ankara, Turkey

**Keywords:** Anemia, hypertension, renin, angiotensin, aldosterone

## Abstract

**Background/aim:**

The relationship between the activation of the renin-angiotensin system and the increase in erythropoiesis has been shown in many studies. In addition, the use of angiotensin converting enzyme inhibitors (ACEIs) or angiotensin-receptor blockers (ARB) has been reported to reduce hemoglobin levels in various patient groups at risk for secondary erythrocytosis/polycythemia. The aim of our study is to investigate whether there is a change in hemoglobin levels after starting ACEIs or ARBs in patients who have not used them before.

**Materials and methods:**

Three hundred and fifty-one patients who were started on renin angiotensin aldosterone system (RAAS) blockers were evaluated retrospectively. None of the patients had anemia before starting RAAS blockers. A median of 6 (4–12) months after the start of the drug, complete blood count and kidney function tests were evaluated. Hemoglobin values before and after the start of the drug were compared statistically.

**Results:**

A statistically significant decrease in mean Hb value was found after starting ACEIs or ARBs (14.39 ± 1.29 g/dL vs 13.98 ± 1.36 g/dL, p < 0.001). The decrease in control Hb values was higher in the ARB group than in the ACEI group (–0.53 ± 0.06 g/dL vs −0.29 ± 0.06 g/dL, p < 0.001).

**Conclusion:**

A significant decrease in mean Hb level was detected in the first year following the first administration of ACEIs or ARBs.

## 1. Introduction

Activation of the renin angiotensin aldosterone system (RAAS) is central to many common pathological conditions such as hypertension, heart failure, and kidney disease. RAAS blockers, which were intended for the treatment of hypertension when they were first used, have since become the first choice in the treatment of hypertension; and independent of their blood pressure lowering effect, RAAS blockers are widely used because of their kidney-protective and heart-protective effects. [1].

Several studies have found that RAAS activation can increase erythropoiesis and be associated with secondary erythrocytosis [2,3]. It has also been reported in the literature that the use of angiotensin converting enzyme inhibitors (ACEIs) or angiotensin receptor blockers (ARBs) reduces hemoglobin (Hb) levels in various patient groups at risk for secondary erythrocytosis/polycythemia [4–6]. In experimental studies, RAAS blockade has been shown to reduce hemoglobin and hematocrit levels [7,8]. The exact mechanism is not fully understood, but angiotensin II may be responsible for erythropoietin secretion or direct stimulation of erythroid progenitors [9,10]. In clinical studies, there are conflicting results as to whether RAAS blockers reduce hemoglobin levels [11,12]. There are studies in the literature reporting that ACEIs have a reducing effect on Hb levels and ARBs have a more limited effect [13], as well as other studies reporting the opposite [14].

The aim of this study is to investigate whether there is a change in Hb levels after starting ACEI or ARB treatment in patients with an estimated glomerular filtration rate (eGFR) ≥30 mL/min and who have not used ACEIs or ARBs before.

## 2. Materials and methods

Adult patients admitted to the nephrology and internal medicine outpatient clinics of the University of Health Sciences-Keçiören Educational and Research Hospital between 01/07/2017 and 01/07/2019 and newly started to use ACEI or ARB group drugs due to diabetic nephropathy or hypertension were included in this retrospective study. Patients with anemia, iron deficiency, vitamin B12 deficiency, folate deficiency, those who were treated for anemia in the last 6 months, and those who had a history of blood transfusion or a history of blood donation in the last 1 year were excluded from the study. Patients who had eGFR less than 30 mL/min/1.73 m^2^, those who had a history of active infection or malignancy, and those who had an operation or any condition that causes active bleeding during study period were also excluded from the study.

The study protocol was approved by the ethics committee of the Keçiören Education and Research Hospital.

Patients’ demographic properties (age, sex, history of diabetes mellitus, history of hypertension, name and group of the newly started antihypertensive drug) were obtained from their medical records. Patients’ laboratory data such as hemogram, serum creatinine, and eGFR levels evaluated before RAAS blockers initiation and at least 3 months after initiation were also obtained from the medical records.

The estimated glomerular filtration rate was calculated by 4-variable MDRD equation described by the National Kidney Foundation as follows: GFR (mL/min/1.73 m^2^) = 175 × (Scr)^−^ −1.154 × (Age)^−^ −0.203 × (0.742 if female) × (1.212 if African American) [15]. Anemia was defined according to WHO criteria that Hb level is below 12 g/dL for women and 13g/dL for men [16].

Data analysis was performed using SPSS version 15.0 (SPSS Inc., Chicago, IL, USA). A *p*-value < 0.05 was considered statistically significant. The Kolmogorov–Smirnov test was used to asses the normality of variables. All continous data showed nonnormal distribution. Results were expressed as median (min–max) for continous variables and as numbers and percent for categorical variables. Chi-square test, Fisher’s exact test, Mann–Whitney *U* test, and unpaired Student’s t-test were used for the comparison of findings in two groups. The values before and after the RAAS began were compared by paired Student’s t-test.

## 3. Results

Three hundred and fifty-one patients were included in the study. The number of patients who started ACEIs and ARBs was 178 and 173, respectively. A complete blood count and kidney function tests were evaluated on average 6 (4–12) months after the start of the drug. The most commonly used ACEIs and ARBs among patients were perindopril (39.8%) and candesartan (48.3%), respectively. Demographic and clinical characteristics of the patients are given in Table 1.

As shown in Table 1, many of the clinical and laboratory characteristics of patients using ACEI and ARB were similar. The percentage of diabetes was higher in the ACEI group (37.6 % vs 20.2%, p < 0.001). It was found that the control blood count was evaluated later after starting the drug in the ARB group than in the ACEI group (7 (4–12) months vs 5 (4–12) months, p < 0.05).

The initial mean Hb value of patients before the drug was started was 14.39 ± 1.29 g/dL. A statistically significant decrease in mean Hb value was found after starting ACEI or ARB (13.98 ± 1.36 g/dL, p < 0.001).

Delta_Hb value (Delta_Hb=control Hb-initial Hb) was obtained by calculating the difference between initial Hb and control Hb. Delta_eGFR (Delta_eGFR=control eGFR-initial eGFR) was obtained by calculating the difference between control eGFR and initial eGFR. It was found that the decrease in control Hb values was greater in the ARB-group than in the ACEI-group (Table 2). There were no significant differences in eGFR changes between the two study groups (Table 2).

## 4. Discussion

In this study, a significant decrease in mean Hb levels was observed in the follow-up of patients who were started on ACEI or ARB. In addition, it was found that the decrease in Hb levels of the patients who were started on ARB was higher than that in the patients who were started on ACEI. Most of the patients included in the study had no renal dysfunction (eGFR ≥ 60 mL/min). However, in a small number of patients with mild renal dysfunction (eGFR 30–60 mL/min), the decrease in mean Hb levels after starting ACEI or ARB was similar to the decrease in mean Hb levels in patients with eGFR ≥ 60 mL/min.

The effects of RAAS on hematopoiesis are observed both during the formation and proliferation of hematopoietic cells in the bone marrow during the embryological period and during the synthesis of erythropoietin and the effect of erythropoietin on target cells. The effect of local RAAS on hematopoietic progenitor cells is thought to be mediated by AT-1 receptors [17,18]. In a study on mice, it was shown that AT-2 deficiency causes anemia, and Hb and hematocrit levels increase with AT-2 infusion [19]. In addition, it was shown that the burst-forming unit (BFU) and colony-forming unit (CFU) levels have increased with the addition of AT-2 to the in vitro culture of human peripheral blood mononuclear cells or bone marrow cells [10,19].

AT-2 has been shown to be associated with erythropoietin production from peritubular fibroblasts of the kidney in vitro [9]. However, there are uncertainties about the relationship between ACE inhibition and erythropoietin levels in vivo. It has been reported in some studies that ACEI administration reduces plasma erythropoietin levels or induces erythropoietin resistance [20,21]. In a study conducted in 10 healthy volunteers in 1992, the possibility of reducing plasma EPO concentrations of enalapril and captopril as ACEIs was investigated. A significant decrease was found in the mean plasma EPO concentration measured after 28 days of treatment with both ACE inhibitors [22]. In addition, when an ACEI, enalapril, was given to hypertensive patients with chronic nephropathy, it was reported in the follow-up after 90 days that the mean Hb value decreased by about 1 g/dL, and EPO levels decreased from 32 U/L to 24 U/L [23]. ACE inhibitors are thought to reduce the formation of EPO by inhibiting the production of angiotensin-II [21,23]. In addition, anemia due to ACEIs and ARBs has been associated with decreased erythropoietin production as a result of increased renal plasma flow and increased oxygen delivery [23]. ACEIs have also been shown to reduce insulin-like growth factor levels. Insulin-like growth factor plays a role in stimulating the erythroid sequence and inhibiting the catabolism of N-acetyl-seryl-aspartyl-proline, a natural peptide that reduces the proliferation of red cell precursor cells [24].

A study of kidney transplant patients concluded that the ACEI-related reduction in Hb levels may reflect modulation of multiple factors interacting with erythroid bone marrow precursors [25]. In parallel, it was reported in an experimental study that losartan, an AT-II type 1 receptor (AT-1) antagonist, developed posttransplant erythrocytosis without altering serum erythropoietin levels [10]. Angiotensin 2 increases the proliferation of early erythroid progenitors, while ARB completely abolishes this effect [10].

Administration of captopril in hemodialysis patients may contribute to worsening of anemia [26]. In addition, it has been shown that the use of ACEI/ARB in patients with end-stage renal disease who need erythropoiesis stimulating agents (ESA) causes ESA resistance and increases the dose of ESA that should be used to correct anemia. [27]. Similarly, both ACEIs and ARBs have been reported to cause ESA resistance in peritoneal dialysis patients [14].

Clinical studies have also investigated the association of RAAS blockers with anemia in patients without significant chronic kidney disease. A retrospective cohort study examined Hb values before and 1 year after initiation of medication in all adults receiving ACEIs, ARBs, or calcium channel blockers. Treatment with ACEIs and ARBs has been found to be associated with a higher risk of anemia and reduced Hb levels. A similar association has not been observed in patients using calcium channel blockers [28]. Another retrospective study investigated whether patients with hypertension, diabetes, or ischemic heart disease had a decrease in Hb levels on average 1 year after initiation of ACEI or ARB. It has been reported that the use of ACEIs is associated with a decrease in Hb levels, and no similar association was found with the use of ARBs [8]. In contrast to that study, Inoue et al. [14] reported a significant decrease in Hb value with ARB use, but this effect was not observed with ACEI in diabetic patients with chronic kidney disease. In our study, a decrease in Hb levels was detected in patients who started both ACEI and ARB, which were evaluated at least 3 months after the start of the drug. However, this decrease in Hb value is more pronounced in ARB users. In our study, there were no significant differences between the sex, age, and basal Hb value between the groups using ACEI and ARB. In addition, patients with severe heart failure were not included in our study to avoid confusion in the results because after starting RAAS blockers in patients with heart failure, it has been reported that there is an increase in Hb levels due to the improvement in heart failure and a decrease in dilution due to congestion.

EPO resistance has been found to increase in patients using ACEIs/ARBs, depending on the dose [14]. In addition, in studies investigating the effects of RAAS blockers on the treatment of secondary polycythemia, a dose-related decrease in hematocrit was reported in the first month of treatment [2,11]. In our study, the effect of dose-related anemia was not compared for reasons such as the absence of standard doses of ACEIs or ARBs and the inability to adequately assess patient compliance due to the retrospective nature of the study. However, the follow-up time for control Hb assessment was higher in the ARB group. Although patient compliance is unknown, a long follow-up period may indicate a high cumulative dose. This may explain the more pronounced decrease in Hb observed with ARBs in our study. In addition, there may be different reasons for the effect of Hb reduction in patients receiving ARBs, which may be the subject of other studies.

The strengths of this study are that Hb levels of all patients included in the study were within normal limits and that parameters such as iron, vitamin B12, and folate levels were evaluated before the patients were started on RAAS blockers. Ferritin, iron/iron binding, vitamin B12, and folate records of the patients at least 6 months before starting the treatment with RAAS blockers were scanned and only patients whose parameters were within the normal range were included in the study. Therefore, only a limited number of patients could be included in the study.

Our study has some limitations. The most important one is the small number of patients included in the study. The number of patients included in the study remained low since only the patients who were retrospectively determined to have met the inclusion criteria were included in the study. The causal relationship between RAAS blockers use and EPO level could not be investigated due to the retrospective nature of the study, as EPO level is not measured routinely. Lastly, there was no standardization regarding the types and doses of ACEIs or ARBs used by the patients included in the study, and the patient compliance could not be fully addressed.

In conclusion, in this study, a significant decrease was detected in the Hb levels measured in the first year following the initial administration of ACEIs or ARBs to patients with hypertension and/or diabetes but without significant renal failure and anemia. This effect was more pronounced in the group that has used ARBs. Although this group of drugs have renoprotective and cardioprotective effects, it may be appropriate to administer to patients with anemia, considering the benefit-harm balance, since anemia may worsen diabetic nephropathy and cardiovascular failure. In addition, it would be more appropriate to evaluate Hb levels in the first year after the first administration of RAAS blockers, especially in patients with a tendency to anemia.

## Figures and Tables

**Table 1:** Comparison of demographic and laboratory characteristics of patients using ACEIs or ARBs.

Number	351	ACEI-group (178)	ARB-group (173)	*p*

Sex (F) (%)	55.6	50.8	49.2	0.981*

Age (years)	56 (19–87)	54 (19–87)	56 (25–79)	0.334**

Diabetes mellitus (%)	26.2	37.6	20.2	0.001*

Hypertension (%)	97.4	94.8	100	0.012*

Follow-up period (months)	6 (4–12)	5 (4–12)	7 (4–12)	0.013**

Hb (g/dL)	14.4 (12.0–16.7)	14.5 (12.0–16.3)	14.3 (12.0–16.7)	0.615**

Htc(%)	43.0 (35.0–53.6)	43.0 (35.0–52.3)	42.0 (36.0–53.6)	0.650**

MCV(fL)	88 (80–100)	88 (82–100)	86 (80–100)	0.776**

RBC(x10⁄μL)	4800 (4200–5600)	4800 (3700–5500)	4700 (3900–5600)	0.939**

Ferritin(ng/mL)	65 (27–277)	47 (28–277)	53 (27–114)	0.626**

Vitamin B12(pg/mL)	323 (198–647)	374 (201–631)	325 (198–647)	0.320**

Folate (ng/mL)	7 (5–17)	7 (5–17)	7 (5–16)	0.530**

Transferrin saturation (%)	36 (20–70)	40 (20–60)	40 (20–70)	0.926**

eGFR (mL/min/1.73 m^2^)	93 (32–127)			
eGFR 30–60 (%)	7.7	93 (34–117)	93 (32–127)	0.988**
eGFR ≥ 60 (%)	92.3			

Serum creatinine (mg/dL)	0.78 (0.30–1.80)	0.77 (0.3–1.80)	0.80 (0.40–1.70)	0.753**

ACEI: Angiotensinogen converting enzyme inhibitors, ARB: Angiotensin receptor blockers, eGFR: Estimated glomerular filtration rate.

* Chi-square test,

** Mann–Whitney *U* test

**Table 2:** Delta_Hb and Delta_eGFR values in study groups.

	ACEI-group (N:178)	ARB-group (N:173)	p*
Delta_Hb	−0.29 ± 0.06	−0.53 ± 0.06	0.001
Delta_eGFR	−3.2 ± 0.80	−1.8 ± 0.77	0.403

ACEI: Angiotensinogen converting enzyme inhibitors, ARB: Angiotensin receptor blockers, eGFR: Estimated glomerular filtration rate.

* Unpaired Student’s t-test
